# Zirconia and Crofer Joint Made by Reactive Air Brazing Using the Silver Base Paste and Cu-Ti Coating Layer

**DOI:** 10.3390/ma17153822

**Published:** 2024-08-02

**Authors:** Shu-Wei Chang, Ren-Kae Shiue, Liang-Wei Huang

**Affiliations:** 1 Department of Materials Science and Engineering, National Taiwan University, Taipei 106, Taiwan; d03527009@ntu.edu.tw; 2 Chemistry and Environment Research Laboratory, Taiwan Power Research Institute, Taiwan Power Company, New Taipei City 238, Taiwan; 3 Department of Material Research, National Atomic Research Institute, Taoyuan 325, Taiwan; i13501350@nari.org.tw

**Keywords:** reactive air brazing, interface, metallization, microstructure, airtight

## Abstract

This study proposes a method to enhance the airtightness of the joint between the ZrO_2_ and Crofer alloy using coating technology. With the aid of vacuum sputtering technology, a titanium–copper alloy layer with a thickness between 1.5 μm and 6 μm was first deposited on the surface of ZrO_2_ and Crofer, respectively. The chemical composition of the deposited reaction layer was 70.2 Cu and 29.8 Ti in at%. Then, using silver as the base material in the reactive air brazing (RAB) process, we explore the use of this material design to improve the microstructure and reaction mechanism of the joint surface between ceramics and metal, compare the effects of different pretreatment thicknesses on the microstructure, and evaluate its effectiveness through air tightness tests. The results show that a coating of Cu-Ti alloy on the ZrO_2_ substrate can significantly improve bonding between the Ag filler and ZrO_2_. The Cu-Ti metallization layer on the ZrO_2_ substrate is beneficial to the RAB. After the brazing process, the coated Cu-Ti layers form suitable reaction interfaces between the filler, the metal, the filler, and the ceramic. In terms of coating layer thickness, the optimized 3 μm coated Cu-Ti alloy layer is achieved from the experiment. Melting and dissolving the Cu-Ti coated layer into the ZrO_2_ substrate results in a defect-free interface between the Ag-rich braze and the ZrO_2_. The air tightness test result shows no leakage under 2 psig at room temperature for 28 h. The pressure condition can still be maintained even under high-temperature conditions of 600 °C for 24 h.

## 1. Introduction

Metals and ceramics are used in different industrial fields due to their specific characteristics. However, in some cases, when taking advantage of two materials at the same time, the two materials must be combined by joining, such as gas turbine blade coatings for power plants [1,2], circuit boards [3,4], biomedical applications [5,6], and fuel cells [7]. Currently known joining technologies for ceramics and metal include brazing, diffusion bonding, and friction welding [8]. Dissimilar metal and ceramic brazing is the primary approach to joining. The braze melt wets the ceramic’s surface, forming a metallurgical bond between the ceramic and metal [9].

The brazing technology can provide a joint with relatively good gas tightness between the ceramic and metal. It can also offer better thermal cycle stability, contributing to the joint’s long-term reliability. It is a sealing approach with potential for industrial applications [10,11,12]. AMB (active metal brazing) and RAB (reactive air brazing) are well-known joining technologies for metal-ceramic sealing. AMB uses an inert gas, such as Ar and He, or a relatively high-vacuum (<10^−8^ bar) in brazing to avoid the deterioration of the metallic filler alloy. Higher production costs are necessary. Moreover, the low oxygen partial pressure needed in the brazing temperature may lead to irreversible degradation of the ceramic substrate [13]. In contrast, RAB is a potential alternative to producing an airtight metal/ceramic joint. It has a lower manufacturing cost without degradation of the ceramic substrate.

ZrO_2_ has been widely applied in SOFCs (solid oxide fuel cells), TBC (Thermal Barrier Coating), and the electronic industry since it is featured with high thermal and chemical stability [7,14,15]. Due to the similar thermal expansion coefficient to ZrO_2_, the Crofer is considered a suitable alloy to join with ZrO_2_. In recent years, a few scholars have proposed using silver as the sealing metal, introducing a few percent oxide to improve its coefficient of thermal expansion mismatch between the Ag and ZrO_2_ [16,17,18]. Some scholars also use low-melting point commercial active solders to join the metal and ceramics [19,20]. It has also been reported that coating technology is used to metalize the surface of ceramics to improve their wettability, allowing brazing to proceed [21,22].

The commercially available Ticusil active braze alloy, 26.7 Cu, 4.5 Ti, and 68.8 Ag in wt%, is a popular filler metal in brazing ZrO_2_ and stainless steel [19,23,24]. However, the oxidation of such a brazed joint deteriorates rapidly because Cu is particularly susceptible to oxidation in high-temperature air environments [25]. Decreasing the Cu amount in the Ag-based braze could be a possible solution to overcome the above issue. Laik et al. [26] used Ag-based commercial active braze alloy to vacuum braze Al_2_O_3_/Inconel 600, which was stable and kept gas-tight and robust. Wang et al. [27] use Ag-5Cu-1Al-1.25Ti (wt%) filler to vacuum braze Al_2_O_3_ and Kovar 4 J34 alloy. Based on these research findings, the feasibility of applying Ag-based filler alloyed with Ti and Cu as brazing material was evidenced to achieve a solid joint for ceramic and metal. Suppose the copper content of the braze alloy can be further reduced through the design of ceramic metallization before the brazing process. In that case, the central issue of silver-based filler may be solved. 

Thin film sputtering coating(s) can be recognized as one of the metallization methods used for ceramic pretreatment before brazing. In a previous study, pure Cu, Ti, and Ag layers were sequentially deposited on the ZrO_2_ substrate, and vacuum brazing of the metalized ZrO_2_ and Crofer alloy using the 72Ag-28Cu (wt%) filler foil was performed [20]. However, the melting points of Cu (1084.87 °C) and Ti (1670 °C) are much higher than that of 70Cu-30Ti in at%, approximately 875 °C [28]. The deposition of the 70Cu-30Ti alloy coating layer on the ZrO_2_ substrate is expected to provide a higher activity in brazing ZrO_2_ and Crofer [19,29,30].

This study proposes a thin film sputtering 70Cu-30Ti (at%) alloy coating layer for ZrO_2_ pretreatment before brazing. Then, it uses the silver paste as the braze filler for the RAB process. This study also discusses how the coating layer design improves the microstructure and reaction mechanism of the ceramic-metal joint and compares different pretreatment thicknesses of the Cu-Ti coating layer on ZrO_2_. Based on the airtightness of the RAB zirconia and Crofer joint, an optimized thickness of the 70Cu-30Ti coating layer is achieved in this study.

## 2. Materials and Experimental Procedures

### 2.1. Materials Preparation

The ZrO_2_ plate was purchased from Kceracell, Geumsan-gun, Republic of Korea. The Crofer plate was purchased from VDM Metals GmbH, Altena, Germany. The chemical composition in wt% is 23 Cr, 0.03 C, 0.8 Mn, 0.2 Si, 0.1 Al, 2.0 W, 0.8 Nb, 0.5 Ni, 0.5 Cu, and Fe balance. ZrO_2_ and Crofer substrates were cut into specimens with 10 × 10 × 1 mm^3^ in dimension. PELCO^®^ colloidal silver was purchased from TED PELLA, Redding, CA, USA. PELCO^®^ colloidal silver with 60 ± 1 wt% Ag, 10~25 wt% toluen, 5~10 wt% ethyl acetate, 5~10wt% ethanol, 5~10 wt% n-butyl acetate, <2.5 wt% propan-2-ol, and <2.5 wt% bornan-2-on.

ZrO_2_ and Crofer substrates were metalized using a sputtering machine (KaoDuen KD-SPA01, New Taipei City, Taiwan). They were preheated to 200 °C under a vacuum of 6 × 10^−6^ mbar, and then argon was introduced to reach a vacuum of about 3 × 10^−2^ mbar. The temperature of the rotating disk was controlled at 200 °C. In the double target Ti (170 W) and Cu (80 W) sputtering machine, the thicknesses of the coating films (1.5 μm, 3 μm, and 6 μm) were controlled by different sputtering periods, 1 h, 2 h, and 4 h. The chemical composition of the coated Cu-Ti alloy layer was 70Cu and 30Ti in at%. An electron microscope (Hitachi S-4800, Tokyo, Japan) and energy dispersive spectrometer (Bruker-Quantax, Berlin, Germany) were used to analyze the chemical composition of the coated layer with 70.2 Cu and 29.8 Ti in at%.

### 2.2. RAB Process

In the RAB, the brazing was performed in a furnace (Ultra Fine UF-E1FS, New Taipei City, Taiwan) at 960 °C with a heating rate of 60 °C/h, held for 20 min, and cooled to 25 °C with a 60 °C/h cooling rate in the air. The cross-section of the brazing specimen was cut with the aid of a slow-speed cutter. The specimen was hot-mounted using the conductive epoxy resin, ground, and polished using a standard metallographic procedure. The sizes of the diamond polishing particles were 9 μm, 3 μm, and 1 μm, respectively. An electron microscope (Hitachi S-4800) and an energy dispersive spectrometer (Bruker-Quantax) were used to analyze the microstructure and phase compositions of the brazed joint. Quantitative chemical analysis was performed using a field emission electron probe microanalyzer (JEOL JXA-8530F Plus, Tokyo, Japan) with a minimum spot size of 1 μm.

### 2.3. Pressure Drop Test

**Figure 1:**
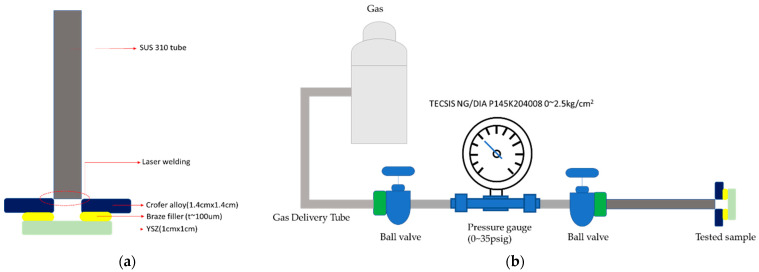
The schematic diagram of the pressure drop test: (**a**) the brazed specimen, (**b**) test infrastructure.

To evaluate the sealing performance of the brazed specimen, a pressure drop method was used to measure the airtightness of the sealed sample [20,21]. Figure 1 shows the schematic diagram of the RAB specimen used in the pressure drop test. The Crofer alloy and ZrO_2_ are 14 × 14 × 1.5 mm^3^ and 20 × 20 × 1 mm^3^. The Crofer substrate has drilled a hole with a diameter of 8 mm in the central area of the Crofer alloy in advance and connected to the gas source through a metal tube, as displayed in Figure 1a. The flow gas was controlled by a ball valve, as shown in Figure 1b. The internal pressure of the brazed specimen to be tested was recorded over time to calculate the gas leakage rate.

## 3. Results and Discussion

**Figure 2:**
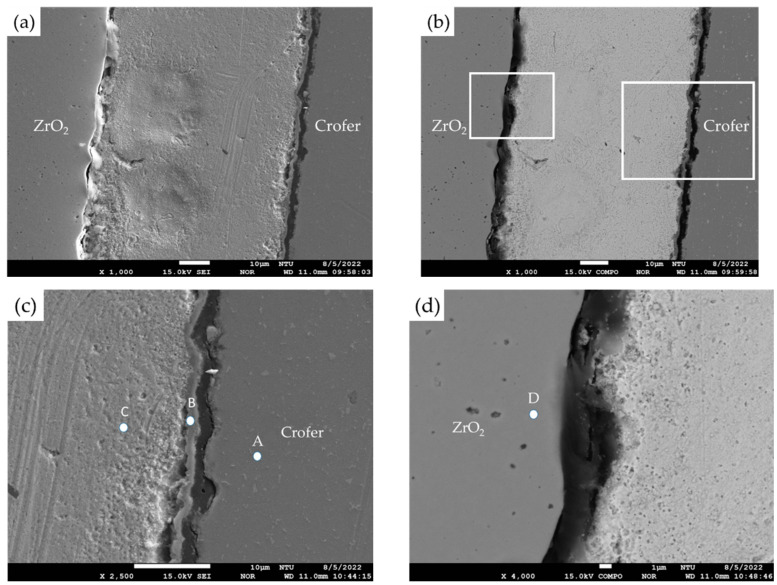
(**a**) SEI, (**b**) BEI of the ZrO_2_/Ag/Crofer joint reactive air brazed at 960 °C for 1200 s; (**c**) higher magnification at the Ag/Crofer interface, (**d**) higher magnification at the ZrO_2_/Ag interface.

For comparison purposes, EPMA microstructural observations and quantitative element analyses were carried out on the cross-section of samples without the titanium–copper coating layer. Both secondary electron image (SEI) and backscattered electron image (BEI) are shown in Figure 2a,b. In Figure 2c,d, interfacial separations were observed among the Ag-rich braze alloy and two substrates, Crofer and ZrO_2_. Table 1 shows WDS quantitative chemical analyses of selected locations of the brazed joint without the Cu-Ti coating layer in Figure 2c,d. In Figure 2c, the Ag-rich matrix and the Crofer substrate were observed, as marked by C and A. According to Table 1, there was a layer of chromium oxide, as marked by B in Figure 2c, and the thickness of the chromium oxide layer on the interface was approximately 1 µm. The stoichiometric ratio between Cr and O at position B was close to Cr_2_O_3_. Figure 2d shows the microstructural observation of the interface between the Ag-rich matrix and the ZrO_2_ substrate. Figure 2d shows an apparent interfacial crack between the ZrO_2_ substrate and the Ag-rich braze. The Ag filler cannot be applied in RAB ZrO_2_ and Crofer alloy due to oxidation of the Crofer substrate in the air and high residual stresses resulting from the mismatch of thermal expansion coefficients between the Ag filler metal and ZrO_2_. An inferior brazed joint with interfacial separations was observed among the Ag filler metal, ZrO_2_, and Crofer substrate.

**Figure 3:**
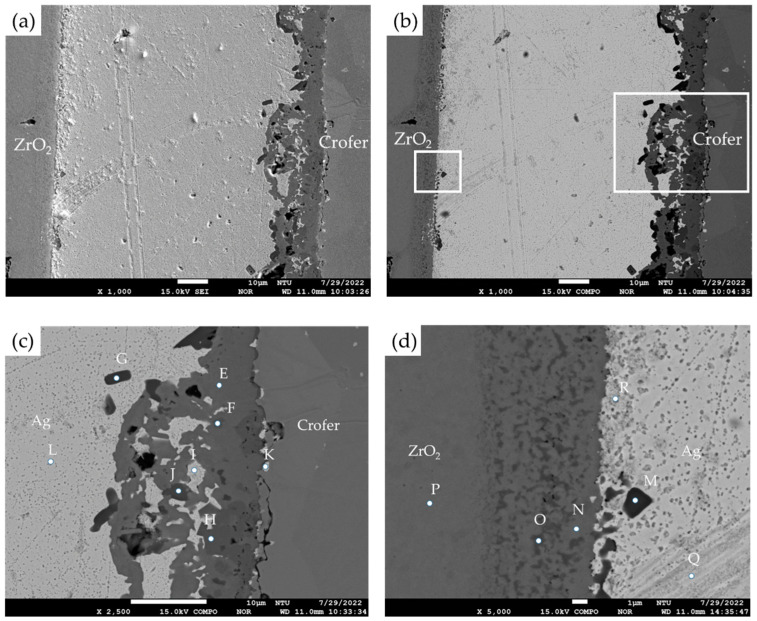
(**a**) SEI, (**b**) BEI of the ZrO_2_/Ag/Crofer joint reactive air brazed with 3 μm coating layer at 960 °C for 1200 s; (**c**) higher magnification at the Ag/Crofer interface, (**d**) higher magnification at the ZrO_2_/Ag interface.

For the RAB joint with a titanium–copper coating layer of 3 μm in thickness on the ZrO_2_ substrate, EPMA microscopic observation and quantitative chemical analyses were carried out, as illustrated in Figure 3 and Table 2. The SEI and BEI of the Crofer/Cu-Ti/ZrO_2_ joint are shown in Figure 3a,b, respectively. The interfaces among the Ag-rich braze and two substrates were significantly improved compared to those in Figure 2a,b, and no separation was observed at the interfaces. Strong interfacial reactions of the coated Cu-Ti layer and ZrO_2_ substrate were observed in Figure 3d. Figure 3c shows the EPMA quantitative chemical analyses of selected positions at the Ag/Crofer interface, and the results are shown in Table 2. In Figure 3c, these oxides primarily alloyed with Cu, Cr, and Fe at positions E, F, and H. TiO_2_ (marked by G) and Fe/Ti-rich oxide (marked by J) were also observed with a darker color. The interfacial reaction layer was mainly composed of oxide, and a few Ag-rich phases (marked by I and K) were dispersed in the oxide layer. The reactive wetting formed a good bond between the coated Cu-Ti layer and ZrO_2_. In Figure 3d, there was an interfacial layer between the Ag-rich matrix (marked by Q and R) and the ZrO_2_ substrate (marked by P), and a Ti-rich oxide was observed as marked by M. The interfacial layer comprised ZrO_2_ alloyed with different Ti/Cu contents, as marked by N and O in Table 2. Dissolving the Cu-Ti coated layer into the ZrO_2_ substrate resulted in a defect-free interface between the Ag-rich braze and substrate, as demonstrated by Figure 3a–d. 

For the RAB joint without the 70Cu-30Ti active coating layer, interfacial separations were extensively observed at the interfaces, as displayed in Figure 2. There was no active ingredient in the braze filler or Ag paste. The wettability of the Ag paste was poor for the ZrO_2_ substrate. Additionally, the wettability of the Ag paste on the oxidized Crofer substrate was also poor during RAB. In contrast, the Cu-Ti alloy coating layer was the only active ingredient during the RAB in the experiment. It formed a Cu/Ti-rich liquid at 960 °C with high activity for both substrates, ZrO_2_ and Crofer [30,31]. The reactive wetting of both substrates was achieved, and the Cu/Ti-rich liquid was reacted and solidified as part of the reaction layers.

The interfacial reaction of the ZrO_2_ side differed from that of the Crofer side, as illustrated in Figure 3. According to the liquidus projection of the Ag-Cu-Ti ternary alloy phase diagram, there were two immiscible liquids (Ag-rich and Cu/Ti-rich) above 900 °C [30,31]. The activity of the Ag-rich liquid was much less than that of the Cu/Ti-rich liquid. The Ag-rich liquid acted as a barrier against the oxidation of the braze alloy during the RAB. The ZrO_2_ substrate was readily wetted by alloying the Cu/Ti liquid, as demonstrated in Figure 3d and Table 2. For the Crofer side, the dissolution of Cr and Fe from the substrate into the Cu/Ti-rich melt and subsequently oxidation during RAB resulted in islands of Cr/Cu/(Fe)-rich oxides away from the interface, instead of an intermediate layer forming directly at the interface, as shown in Figure 3c and Table 2. The Cr-O binary alloy phase diagram indicates that Cr_2_O_3_ is the only oxide [28]. The formation of Cr_2_O_3_ in Figure 2c without the 70Cu-30Ti coating layer was detrimental to the bonding between the Ag filler and Crofer substrate. Separation of the Cr_2_O_3_ and Crofer substrate was observed in Figure 2c. In contrast, Cr/Cu/(Fe)-rich oxides formed in the interface with 3 μm 70Cu-30Ti active coating layer after RAB was free of crack, as shown in Figure 3c.

The oxidation of the interface for the Crofer side was much more vigorous than that of the ZrO_2_ side. The thickness of the interfacial reaction layer on the Crofer side was approximately between 10 and 20 μm. In contrast, the interfacial thickness of the ZrO_2_ side was approximately 5 μm.

**Figure 4:**
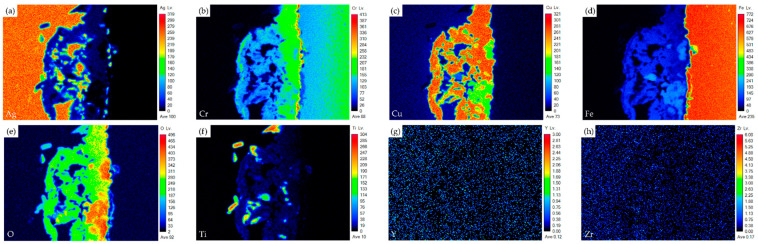
EPMA element mappings of the Crofer side interface in Figure 3c: (**a**) Ag, (**b**) Cr, (**c**) Cu, (**d**) Fe, (**e**) O, (**f**) Ti, (**g**) Y, and (**h**) Zr.

**Figure 5:**
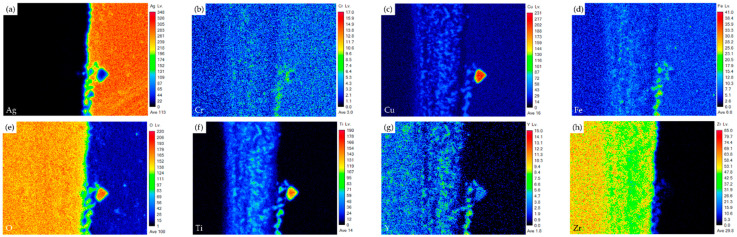
EPMA element mappings of the ZrO_2_ side interface in Figure 3d: (**a**) Ag, (**b**) Cr, (**c**) Cu, (**d**) Fe, (**e**) O, (**f**) Ti, (**g**) Y, and (**h**) Zr.

Figure 4 shows the EPMA element mappings of the Crofer side in Figure 3c. The interfacial oxide layer mainly comprises Cr, Cu, and Fe. The Ti-rich oxide particles are dispersed in the oxide layer. The Cr element is enriched at the interface close to the Crofer substrate, and oxidation of the Crofer substrate is observed after RAB. The EPMA element mappings at the interface between the Ag braze and ZrO_2_ substrate in Figure 3d are shown in Figure 5. Unlike the interface of the Crofer side displayed in Figure 4, the distribution of Cu, O, and Ti agrees with the quantitative chemical analysis results shown in Table 2. The Cu/Ti-rich melt strongly reacts with the ZrO_2_ substrate and promotes reactive wetting of the ZrO_2_ substrate [19]. Both EPMA mappings are consistent with the aforementioned quantitative chemical analysis results. The Cu-Ti metallization layer on the ZrO_2_ substrate is beneficial to the RAB.

**Figure 6:**
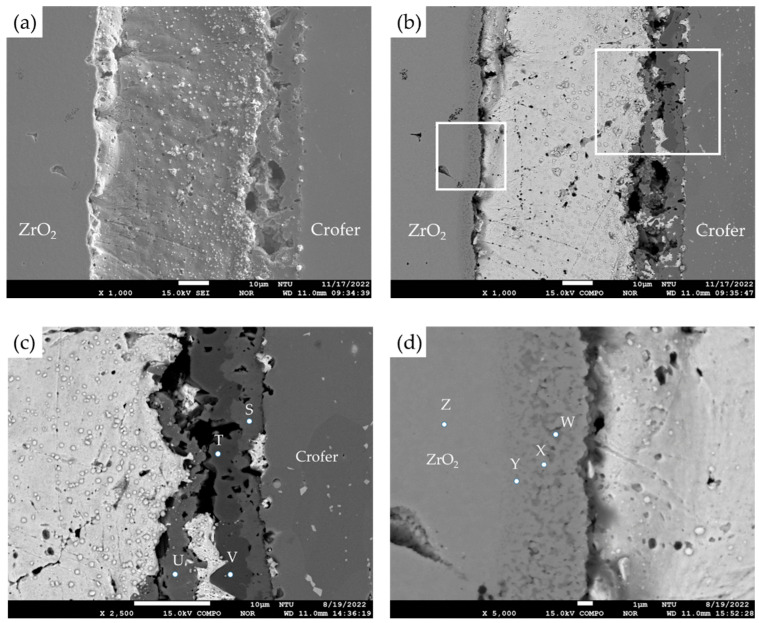
(**a**) SEI, (**b**) BEI of the ZrO_2_/Ag/Crofer joint brazed with 1.5 μm coated film at 960 °C for 1200 s, (**c**) higher magnification at the Ag/Crofer interface, (**d**) higher magnification at the ZrO_2_/Ag interface.

To evaluate the effect of Cu-Ti coated layer thickness on the RAB joints, RAB was conducted with different Cu-Ti coating thicknesses. Both SEI and BEI of the RAB specimen with a 1.5 μm Cu-Ti layer thickness are shown in Figure 6. Under the condition of a 1.5 μm Cu-Ti coating layer, the thickness of the interfacial reaction layer between the Ag-rich braze and ZrO_2_ is significantly decreased to approximately 5 μm, as displayed in Figure 6d. However, there are many pores and cracks, as shown in Figure 6a–c. It is expected that the RAB joint with a 1.5 μm Cu-Ti coating layer on the substrate is not gas-tight. The microstructure of the interface between the Ag braze and ZrO_2_ is shown in Figure 6d. Similarly, the thickness of the interface in Figure 6d is much less than that of 3d. 

Table 3 shows EPMA WDS quantitative chemical analyses of selected positions in the RAB joint with a 1.5 µm Cu-Ti coated layer in Figure 6c and Figure 6d, respectively. The 1.5 µm Cu-Ti coated layer on the Crofer substrate was alloyed with Cr and Fe, and was oxidized into Cr/Cu/(Fe)-rich oxide as marked by S, T, U, and V in Figure 6c. For the ZrO_2_ side of the RAB joint, the ZrO_2_ substrate close to the interface was alloyed with Ti and Cu, as marked by W, X, and Y in Figure 6d. Cu and Ti concentrations decreased with increasing distance from the braze/ZrO_2_ interface. 

**Figure 7:**
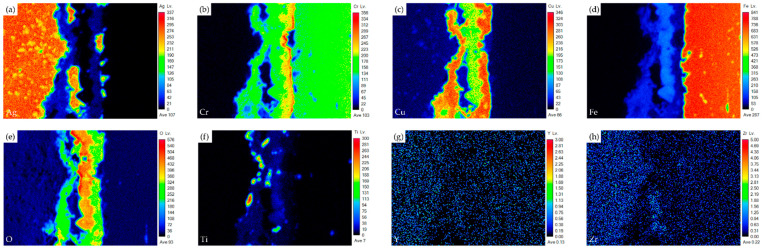
EPMA element mappings of the Crofer side interface in Figure 6c: (**a**) Ag, (**b**) Cr, (**c**) Cu, (**d**) Fe, (**e**) O, (**f**) Ti, (**g**) Y, and (**h**) Zr.

**Figure 8:**
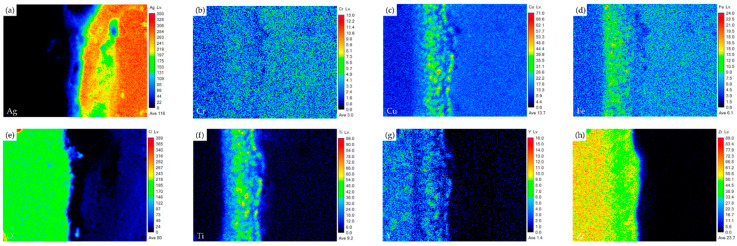
EPMA element mappings of the ZrO_2_ side interface in Figure 6d: (**a**) Ag, (**b**) Cr, (**c**) Cu, (**d**) Fe, (**e**) O, (**f**) Ti, (**g**) Y, and (**h**) Zr.

Figure 7 shows EPMA element mappings of the Crofer side in Figure 6c. The interfacial oxide layer mainly comprises Cr, Cu, and Fe. Less Ti-rich oxide particles are observed in the oxide layer. The Cr element is enriched at the interface close to the Crofer substrate, and oxidation of the Crofer substrate is observed after RAB. The EPMA element mappings at the interface between the Ag-rich braze and ZrO_2_ substrate (Figure 6d) are shown in Figure 8. It has been reported that The Cu/Ti-rich melt has a strong reaction with the ZrO_2_ substrate and promotes reactive wetting of the ZrO_2_ substrate. Both EPMA mappings are consistent with the aforementioned quantitative chemical analysis results.

Strong interfacial reaction and oxidation were observed in the RAB with the 1.5 μm Cu-Ti coating layer. However, thinner reaction/oxidation interfacial layers were observed compared to those of the 3 μm Cu-Ti coating layer due to less Cu and Ti deposited on the substrate. The RAB joint has many microcracks and pores with the 1.5 μm Cu-Ti coating layer, as illustrated in Figure 6. Because the interfacial reaction/oxidation layers were prone to fracture in the joint with a 1.5 μm Cu-Ti coating layer, the airtightness of the RAB joint is inferior to that of the joint with a 3 μm Cu-Ti coating layer.

**Figure 9:**
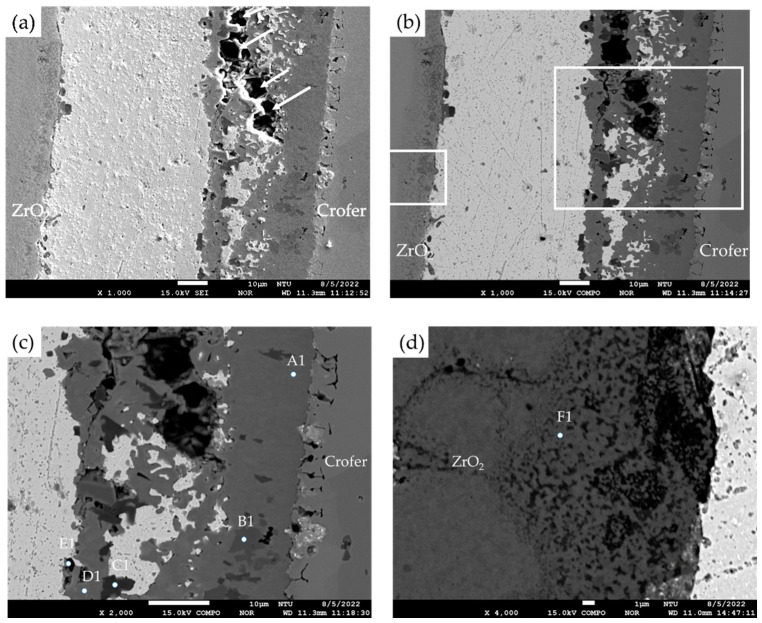
(**a**) SEI, (**b**) BEI of the ZrO_2_/Ag/Crofer joint brazed with 6 μm coated film at 960 °C for 1200 s; (**c**) higher magnification at the Ag/Crofer interface, (**d**) higher magnification at the ZrO_2_/Ag interface.

Figure 9 shows an RAB joint’s SEI and BEI images with a 6 μm Cu-Ti coating layer thickness. The thickness of the interfacial reaction layer on the Crofer side was significantly increased to approximately 30 μm, as illustrated in Figure 9a,b. The Cu-Ti alloy coating formed a Cu/Ti-rich liquid with high activity for both substrates, ZrO_2_ and Crofer. The Cu/Ti-rich liquid was reacted and oxidized as part of the reaction layers next to the substrates, as displayed in Figure 9. It was noted that pores were in the interfacial reaction layer, as indicated by arrows in Figure 9a. The maximum size of the pore was approximately 10 μm. 

The microstructure and EPMA quantitative chemical analysis results of selected positions at the interface of the Crofer side are shown in Figure 9c and Table 4. Regarding chemical composition, the interfacial layer is mainly composed of oxides. There are different oxide particles in the thick Cr/Cu/(Fe)-rich oxide layer, as marked by A1~E1 in Figure 9c. The microstructure and EPMA quantitative chemical analysis results of the ZrO_2_ side are shown in Figure 9d and Table 4. The connection between the Ag-rich braze alloy and ZrO_2_ is in good shape with regards to the microstructure. The ZrO_2_ substrate close to the interface is alloyed with Ti, as marked by F1 in Figure 9d and Table 4. The thickness of both interfacial reaction layers in the RAB with the 6 μm Cu-Ti coating layer is much greater than in the previous cases. 

**Figure 10:**
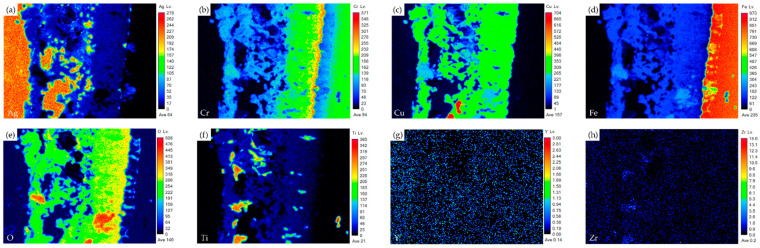
EPMA element mappings of the Crofer side interface in Figure 9c: (**a**) Ag, (**b**) Cr, (**c**) Cu, (**d**) Fe, (**e**) O, (**f**) Ti, (**g**) Y, and (**h**) Zr.

**Figure 11:**
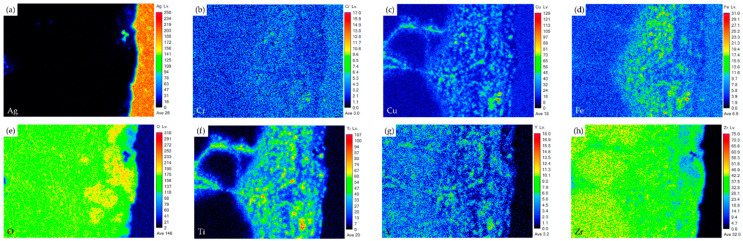
EPMA element mappings of the ZrO_2_ side interface in Figure 9d: (**a**) Ag, (**b**) Cr, (**c**) Cu, (**d**) Fe, (**e**) O, (**f**) Ti, (**g**) Y, and (**h**) Zr.

Figure 10 shows the EPMA element mappings of the RAB joint with a 6 μm Cu-Ti coating layer at the interface of the Crofer side. Under the 6 μm film thickness, oxidation of the Cu-Ti coating layer and Crofer substrate is easily identified. Because the Cu-Ti coating film was thicker, the thickness of the interfacial oxide layer at the Crofer side was increased to nearly 30 μm. The interfacial defects at the Crofer side significantly deteriorated the gas-tight performance of the RAB joint. The EPMA element mappings of the interface at the ZrO_2_ side are shown in Figure 11. Unlike the Crofer side in Figure 10, the diffusion of copper and titanium elements in the interface layer is slightly different. The concentration of titanium in the interface layer is relatively low. The diffusion depth of titanium and copper elements can reach up to 20 µm, while silver elements do not diffuse into the ceramics.

**Figure 12:**
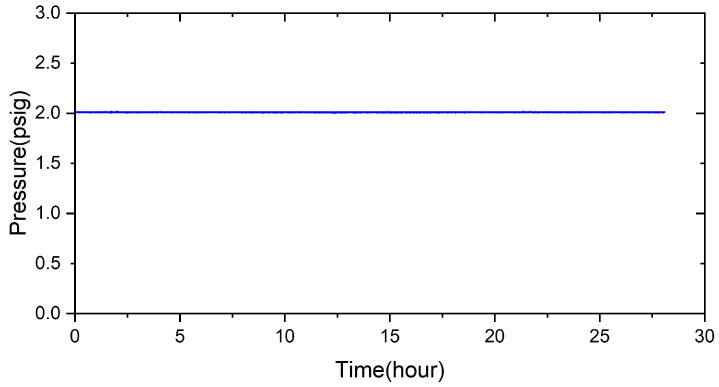
The pressure drop in the joint leak test was performed at room temperature for 28 h.

**Figure 13:**
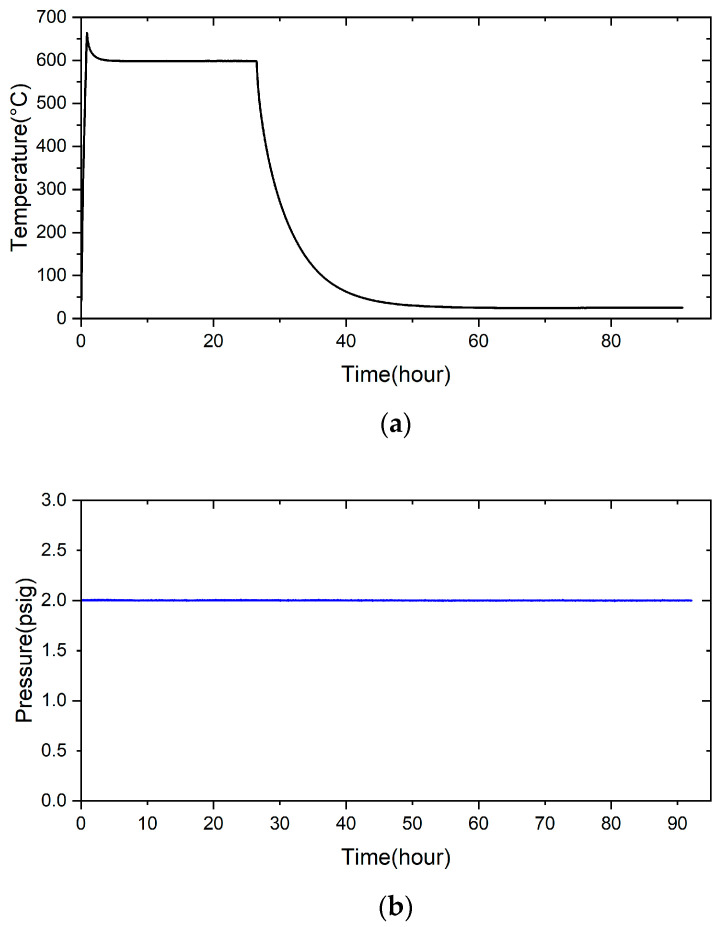
The pressure drop in the joint leak test was at 600 °C for 24 h: (**a**) temperature and (**b**) pressure profiles.

To evaluate the sealing performance of the brazed specimen, a pressure drop test was used to measure the airtightness of the sealed sample. According to the airtight test method, the 3 μm brazed samples were subjected to airtightness tests at room and high temperatures. The test condition was 2 psig helium and room temperature for 28 h, as shown in Figure 12. The test result shows that the pressure did not drop at room temperature. The same test condition was changed to a high temperature of 600 °C, and the result is shown in Figure 13, which also does not show the pressure drop. Applying a 70Cu-30Ti (at%) alloy coating layer on two substrates before reactive air brazing contributes to excellent airtight performance of the joint.

## 4. Conclusions

A novel method for reactive air brazing at 960 °C for the 1200 s to join Cu-Ti metalized ZrO_2_ and Crofer alloy using Ag paste as the braze alloy has been proposed, which proved effective in achieving airtightness of the joint. Important conclusions are summarized below:

1. Applying 70Cu-30Ti (at%) active coating layer on both substrates can effectively improve the bonding between pure silver and ZrO_2_ and pure silver and Crofer alloy.
2. The interfacial reaction of the ZrO_2_ side is very different from that of the Crofer side. There are two immiscible liquids, Ag-rich and Cu/Ti-rich liquids, at 960 °C. The activity of the Ag-rich liquid is much less than that of the Cu/Ti-rich liquid. The Ag-rich liquid acts as a barrier against the oxidation of the braze alloy during the RAB. The ZrO_2_ is readily wetted by alloying the Cu/Ti liquid. For the Crofer side, the Cu/Ti-rich liquid reacted with Fe and Cr in the Crofer substrate and oxidized during the RAB.
3. The oxidation of the interface for the Crofer side was more vigorous than that of the ZrO_2_ side. The thickness of the interfacial reaction layer on the Crofer side was approximately 10 and 20 μm. In contrast, the interfacial thickness of the ZrO_2_ side was approximately 5 μm.
4. The coating thickness of the 70Cu-30Ti active layer is optimized into 3 μm in the experiment. ZrO_2_/Ag and Ag/Crofer interfaces are free of pores and cracks.
5. There is no pressure drop under 2 psig at room temperature for 28 h. The pressure condition can still be maintained even under the high-temperature condition of 600 °C for 24 h. It can potentially be used in solid oxide fuel cells in the future.

## Figures and Tables

**Table 1:** EPMA quantitative chemical analyses of the reactive air-brazed joint without coating in Figure 2c,d.

Element/at%	Ag	Cu	Cr	Fe	O	Ti	Y	Zr	Alloy/Phase
A	0.0	0.0	23.9	73.8	0.00	0.0	0.0	0.0	Crofer (substrate)
B	1.2	0.0	35.0	2.1	59.2	0.0	0.0	0.0	Cr_2_O_3_
C	97.6	0.0	0.5	1.0	0.5	0.0	0.0	0.0	Ag-rich
D	0.0	0.0	0.0	0.0	63.3	0.0	2.0	34.6	ZrO_2_ (substrate)

**Table 2:** EPMA quantitative chemical analyses of the reactive air-brazed joint with a 3 µm Cu-Ti coating layer in Figure 3c,d.

Element/at%	Ag	Cu	Cr	Fe	O	Ti	Y	Zr	Alloy/Phase
Crofer	0.0	0.1	16.8	81.2	0.0	0.0	0.0	0.0	Crofer substrate
E	1.7	23.5	19.6	4.2	49.7	0.8	0.0	0.0	Cr/Cu-rich oxide
F	0.4	14.3	13.9	11.1	57.0	1.4	0.0	0.0	Cr/Cu/Fe-rich oxide
G	2.2	0.0	0.6	0.5	64.5	31.3	0.0	0.0	TiO_2_
H	0.2	13.3	12.7	13.3	57.8	1.5	0.0	0.0	Cr/Cu/Fe-rich oxide
I	94.5	2.2	0.8	0.9	1.2	0.1	0.0	0.0	Ag-rich
J	0.5	4.5	1.7	16.7	57.6	18.5	0.0	0.0	Fe/Ti-rich Oxide
K	81.2	2.7	4.6	8.4	2.2	0.1	0.0	0.0	Ag-rich
L	99.6	0.0	0.0	0.0	0.1	0.1	0.0	0.0	Ag-rich
M	3.4	14.6	0.2	0.0	58.4	20.3	2.5	0.4	Ti-rich oxide
N	0.1	0.3	0.0	0.0	63.8	3.1	0.6	32.1	ZrO_2_ alloyed with Ti
O	0.1	2.7	0.2	0.0	62.5	8.5	3.5	21.8	ZrO_2_ alloyed with Ti/Cu
P	0.0	0.0	0.0	0.0	64.3	0.1	1.9	33.7	ZrO_2_ substrate
Q	99.6	0.0	0.0	0.0	0.2	0.0	0.0	0.0	Ag-rich
R	93.3	0.2	0.0	0.1	1.1	0.2	0.0	4.5	Ag-rich

**Table 3:** EPMA quantitative chemical analyses of the brazed joint with a 1.5 µm Cu-Ti coated layer in Figure 6c,d.

Element/at%	Ag	Cu	Cr	Fe	O	Ti	Y	Zr	Phase/Alloy
S	6.4	37.9	28.7	5.2	20.5	0.8	0.0	0.0	Cr/Cu-rich Oxide
T	0.3	25.4	22.1	19.7	27.7	2.4	0.0	0.0	Cr/Cu/Fe-rich Oxide
U	8.9	37.4	19.9	10.3	20.9	1.6	0.0	0.0	Cr/Cu/Fe-rich Oxide
V	0.5	25.8	21.4	19.1	27.2	2.9	0.0	0.0	Cr/Cu/Fe-rich Oxide
W	0.1	3.6	0.0	0.7	61.6	10.6	4.8	17.6	ZrO_2_ alloyed with Ti/Cu
X	0.1	0.6	0.0	0.1	64.9	3.9	1.3	28.7	ZrO_2_ alloyed with Ti
Y	0.1	0.4	0.0	0.3	64.8	2.9	0.9	30.5	ZrO_2_ alloyed with Ti
Z	0.0	0.0	0.0	0.0	62.4	0.0	3.2	34.3	ZrO_2_ (substrate)

**Table 4:** EPMA quantitative chemical analyses of junction between 6 µm film brazing sample and Crofer in Figure 9.

Element/at%	Ag	Cu	Cr	Fe	O	Ti	Y	Zr	Alloy/Phase
A1	1.9	24.2	19.3	5.9	47.3	0.9	0.0	0.0	Cr/Cu-rich Oxide
B1	0.1	15.3	10.4	15.7	55.5	1.9	0.0	0.0	Cr/Cu/Fe-rich Oxide
C1	0.7	0.3	0.8	1.4	62.4	32.2	0.0	0.0	TiO_2_
D1	4.6	22.8	8.3	10.3	50.5	3.2	0.0	0.0	Cr/Cu/Fe-rich Oxide
E1	1.2	0.2	0.6	1.1	62.8	32.4	0.0	0.0	TiO_2_
F1	0.0	0.4	0.0	0.0	64.9	3.2	1.0	29.9	ZrO_2_ alloyed with Ti

## Data Availability

The raw data supporting the conclusions of this article will be made available by the authors on request.
